# Neuroimaging Evidence of Acupuncture Effects in Parkinson’s Disease: A Systematic Review of fMRI-Based Clinical Studies

**DOI:** 10.3390/healthcare14203457

**Published:** 2026-10-15

**Authors:** Catarina Ramos Pereira, Henry Johannes Greten, Rubim Santos, Ana Mafalda Reis, Bruno Ramos, Maria João Santos, Jorge Machado, Fabrício Pereira, Natália M. de Oliveira, Maria Begoña Criado

**Affiliations:** 1 ICBAS—Abel Salazar Institute for Biomedical Sciences, University of Porto, 4099-002 Porto, Portugal; 2 Piaget Institute, Vila Nova de Gaia, 4099-002 Porto, Portugal; 3 CBSIn—Center of Biosciences in Integrative Health, 4099-002 Porto, Portugal; 4 Academia de Saúde C+, 4099-002 Porto, Portugal; 5 DGTCM—German Society of Traditional Chinese Medicine, 69126 Heidelberg, Germany; 6 HSCM—Heidelberg School of Chinese Medicine, 69126 Heidelberg, Germany; 7 ESS, Polytechnic of Porto, 4099-002 Porto, Portugal; 8 Hospital Pedro Hispano, 4464-513 Matosinhos, Portugal; 9 Department of Radiology and Medical Imaging, Centre Hospitalier Universitaire de Nîmes, 30000 Nîmes, France; 10 Associate Laboratory i4HB—Institute for Health and Bioeconomy, IUCS-CESPU, 4585-116 Gandra, Portugal; 11 Toxicologic Pathology Research Laboratory, 1H-TOXRUN, UCIBIO—Applied Molecular Biosciences Unit, IUCS-CESPU, 4585-116 Gandra, Portugal

**Keywords:** functional magnetic resonance imaging, Parkinson’s disease, acupuncture, neuroimaging, systematic review

## Abstract

**Background/Objectives:** Parkinson’s disease is the second most prevalent neurodegenerative disorder, and many patients seek adjunctive non-pharmacological treatments such as acupuncture, whose neural mechanisms remain poorly characterized. Functional magnetic resonance imaging (fMRI) allows those mechanisms to be examined in vivo. We systematically synthesized the available fMRI evidence on the neural effects of acupuncture in Parkinson’s disease and appraised the methodological quality of that evidence. **Methods:** EMBASE, MEDLINE, PubMed, ScienceDirect, The Cochrane Library, CENTRAL and SciELO were searched, with no restriction on year of publication. Eligible studies were clinical fMRI studies of acupuncture in patients with Parkinson’s disease, of any interventional or controlled cross-sectional design, published in English or Portuguese. Screening and data extraction were performed independently by two reviewers using Rayyan. Risk of bias was assessed with RoB 2 for studies with a randomized element and with ROBINS-I for non-randomized studies. **Results:** Seven studies, reported in eight publications and published between 2009 and 2024, were included, comprising 151 patients with Parkinson’s disease (147 contributing analysable data) and 36 healthy controls, with sample sizes from 8 to 74 participants. One was a three-arm randomized controlled trial, one was a random-order crossover placebo study, one a non-randomized two-group study, two were single-arm interventional studies, and two were controlled cross-sectional comparisons. GB34 was used in five of the seven studies, and all used 3-Tesla echo-planar BOLD imaging. Acupuncture was consistently associated with modulation of the precentral gyrus, prefrontal cortex, thalamus, cerebellum, insula and putamen, accompanied in individual studies by improvements in pain, gait or motor scores. Risk of bias was high in both studies with a randomized element and serious or critical in the five non-randomized studies; no study was at low risk of bias. **Conclusions:** These findings are preliminary and hypothesis-generating. They are not sufficient to establish clinical efficacy, to separate specific neuromodulatory effects from non-specific ones, or to support a change in clinical practice. Adequately powered, sham-controlled randomized trials with harmonized imaging protocols are required.

## 1. Introduction

Parkinson’s disease is the second most prevalent neurodegenerative disorder, and its prevalence is expected to double over the coming decades. Even under optimal dopaminergic management, patients experience progressive decline in mobility, in the performance of daily activities, and in social participation, with a consequent loss of autonomy and quality of life. There is still no definitive diagnostic test: diagnosis rests on clinical criteria supported by magnetic resonance imaging, which serves principally to distinguish Parkinson’s disease from atypical parkinsonian syndromes [1,2], and identification of the earliest stages of the disease remains a major unmet need [3]. The therapeutic gap is equally clear. Available treatments are symptomatic, their benefit wanes as the disease progresses, and their adverse effects accumulate.

It is partly for these reasons that a large proportion of patients with Parkinson’s disease turn to complementary treatments, with acupuncture being the most frequently chosen: 61% of patients surveyed in Singapore and 76% of those surveyed in Korea reported such use [4,5]. The corresponding clinical literature is substantial. Randomized trials and systematic reviews have reported effects of acupuncture on motor scores, gait, pain, sleep and quality of life in Parkinson’s disease, and meta-analyses of traditional Chinese medicine interventions have reported benefits for non-motor symptoms [6]. That literature is, however, methodologically weak. In a scoping review of 23 systematic reviews covering 242 primary studies, only 18.6% of the primary studies were rated as high quality and 43.8% as low quality, and 22 of the 23 reviews were themselves rated as very low quality by AMSTAR 2, leading the authors to conclude that firm conclusions on the efficacy of acupuncture in Parkinson’s disease cannot yet be drawn [7]. Two problems recur across that literature: randomization and allocation procedures are inadequately reported, and the mechanism of any observed effect is almost never investigated.

The mechanistic question is, however, tractable. Parkinson’s disease is characterized by dysfunction of identifiable circuits—the nigrostriatal and wider basal ganglia pathways, and the cerebello-thalamo-cortical circuit—and functional magnetic resonance imaging measures activity and connectivity within those circuits non-invasively and without ionizing radiation [8]. If acupuncture acts through a specific neural mechanism rather than through expectancy alone, that mechanism should be detectable as modulation of these circuits and should covary with clinical change. Functional MRI therefore offers what the clinical trial literature has largely lacked: an outcome that is not reported by the participant and cannot be shaped by their beliefs about the treatment. Several studies have used fMRI to examine the cerebral response to acupuncture in Parkinson’s disease, but they differ widely in design, acupoint prescription, analysis pipeline and clinical outcome measures, and no synthesis has yet brought them together.

The aim of this review was to synthesize the available functional MRI evidence on the neural effects of acupuncture in Parkinson’s disease.

Three objectives were defined. First, to identify which brain regions and networks are consistently modulated by acupuncture in patients with Parkinson’s disease. Second, to determine whether these neurofunctional changes are accompanied by measurable changes in motor, sensory or cognitive clinical outcomes. Third, to appraise the methodological quality of the available evidence and to define the design requirements for future confirmatory trials.

No quantitative synthesis was attempted, for the reasons set out in Section 2 and Section 4.3.

## 2. Materials and Methods

The review followed the Cochrane Handbook [9] and the Preferred Reporting Items for Systematic Reviews and Meta-Analyses (PRISMA) 2020 statement [10].

### 2.1. Search Strategy and Study Identification

An initial systematic search was conducted between 1 March and 30 April 2025 in EMBASE, MEDLINE, PubMed, ScienceDirect, The Cochrane Library, the Cochrane Central Register of Controlled Trials (CENTRAL) and SciELO, combining terms for Parkinson’s disease, functional magnetic resonance imaging and acupuncture. That search was restricted to randomized controlled trials published between 2013 and 2023.

During peer review, the eligibility criteria were broadened, for the reasons given in Section 2.2: restricting the evidence to randomized controlled trials was not appropriate for a mechanistic question addressed predominantly by non-randomized imaging designs. Because the design and date filters of the initial search were no longer consistent with the eligibility criteria, the search was repeated on 15 September 2026 without any restriction on publication type or year of publication, and with an expanded set of controlled vocabulary and free-text terms. Complete database-specific strategies, including field tags, filters, dates of execution and the number of records retrieved from each source, are reported in Supplementary Table S1.

The reference lists of all included reports and of relevant reviews were additionally searched by hand. Studies identified in this way are reported separately in the flow diagram, in accordance with PRISMA 2020.

Two restrictions are acknowledged. First, EMBASE could not be searched directly, as no institutional access was available; coverage of EMBASE-indexed records was partial, through CENTRAL. Second, eligibility was restricted to reports published in English or Portuguese, as the review team could not guarantee reliable data extraction from other languages; this may have excluded relevant Chinese-language reports, which are numerous in this field. Both restrictions are listed among the limitations in Section 4.3.

This review was not prospectively registered. The protocol was defined a priori by the review team and is available from the corresponding author on request. The absence of prospective registration is acknowledged as a limitation in Section 4.3.

### 2.2. Eligibility Criteria

Eligibility was defined a priori as follows.

• Population: Adults with a diagnosis of idiopathic Parkinson’s disease established by a neurologist, without restriction as to age, sex, or disease duration. Studies of parkinsonian syndromes, atypical parkinsonism, or those of patients with severe intercurrent complications were excluded, as were studies in animals, in vitro studies, and studies conducted exclusively in healthy volunteers.
• Intervention: Any acupuncture modality within the framework of traditional Chinese medicine, including manual acupuncture, electroacupuncture and scalp acupuncture, irrespective of acupoint prescription, needling technique, session frequency or treatment duration. Auricular acupuncture, Tui Na, Chinese herbal medicine, Qi Gong, moxibustion and pharmacopuncture were not eligible interventions, nor were interventions combining acupuncture with a herbal co-intervention in a way that prevented attribution of the effect to needling.
• Comparator: No restriction. Sham or placebo acupuncture, no treatment, waiting list, tactile stimulation, a within-participant pre-intervention condition and healthy control groups were all acceptable.
• Outcome: Cerebral activity or functional connectivity measured by any functional magnetic resonance imaging modality, whether task-based or resting-state. Studies whose only imaging outcome was structural, and studies using other neuroimaging modalities alone, were excluded. Clinical outcomes were extracted where reported but were not required for inclusion.
• Study design: Randomized controlled trials, randomized crossover studies, non-randomized controlled trials, single-arm pre–post interventional studies, and controlled cross-sectional fMRI comparisons were all eligible. Case reports, narrative and systematic reviews, conference abstracts and study protocols without results were excluded. When two or more reports described the same cohort, they were treated as a single study and data were extracted from all of them.
• Publication: Reports published in English or Portuguese, with no restriction on year of publication.

### 2.3. Data Extraction and Risk-of-Bias Assessment

Records were screened, and data were extracted independently by two reviewers (N.M.O. and M.B.C.) using the Rayyan platform, with a third reviewer (C.R.P.) available for arbitration. The following were extracted: study identifiers (authors, year, country, journal); participants (eligibility criteria, diagnostic criteria, number enrolled and number analyzed, age, sex, disease duration and baseline severity); study methods (design, allocation and concealment procedures, blinding, registration); intervention characteristics (acupoints, needling parameters, session frequency and duration); imaging protocol (scanner, sequence, analysis pipeline, statistical thresholding); and outcomes.

In the version of this manuscript first submitted, this section stated that methodological quality had been assessed with the Cochrane risk-of-bias tool. That statement overstated what had been done: the included studies had been appraised critically by the review team, but a formal, domain-level risk-of-bias assessment had not been documented, and no study-level results were available to report. We are grateful to the reviewers for identifying this discrepancy.

A complete assessment has now been conducted and is reported in full. Because the included studies are of mixed design, a single instrument was not appropriate. The two studies with a randomized element were assessed with RoB 2 [11], using the crossover extension where applicable; the five non-randomized studies were assessed with ROBINS-I [12]. Each study was assessed independently by two reviewers (N.M.O. and M.B.C.), domain by domain, with disagreements resolved by discussion and, where necessary, by arbitration (C.R.P.). Per-study, per-domain judgements with supporting justifications are summarized in Section 3.7, and are carried into the interpretation of the findings in Section 4.

## 3. Results

### 3.1. Study Selection

**Figure 1:**
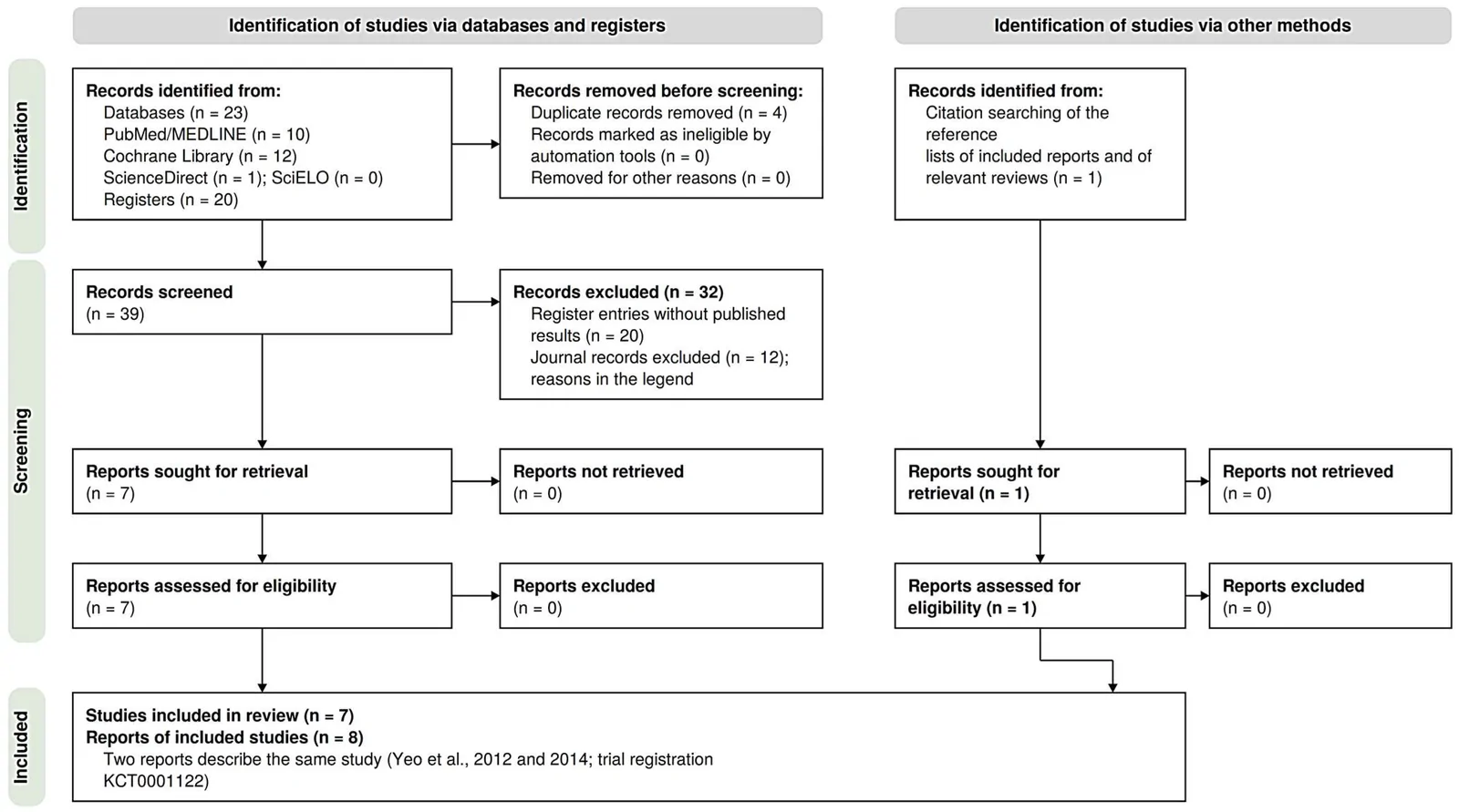
Flow diagram of the study selection process, adapted from the PRISMA 2020 statement [10]. Of the 32 records excluded at title and abstract stage, 20 were trial-register entries for which no results had been published, 4 were study protocols without results, 3 were conference abstracts, 2 were reviews, 1 was an editorial comment, 1 was an animal study, and 1 reported acupuncture combined with a herbal co-intervention and structural imaging only. Two of the eight included reports describe the same study [13,14], so the diagram distinguishes studies from reports of included studies. In the citation-searching arm, n denotes the number of reports identified as potentially eligible; the number of individual citations screened by hand was not recorded prospectively, and this is acknowledged among the limitations in Section 4.3.

The search identified 43 reports, 23 from bibliographic databases and 20 from trial registers accessed through CENTRAL. Four were duplicates retrieved from more than one source, leaving 39 records for screening. Thirty-two were excluded at the title and abstract stage: 20 trial-register entries for which no results had been published, and 12 journal records—four study protocols without results, three conference abstracts, two reviews, one editorial comment, one animal study, and one study in which acupuncture was combined with a herbal co-intervention and only structural imaging was reported. Seven reports were sought for retrieval; all were obtained, and all met the eligibility criteria. One further study was identified by hand-searching reference lists. The selection process is summarized in Figure 1.

### 3.2. Characteristics of the Included Studies

Seven studies, reported in eight publications and published between 2009 and 2024, met the eligibility criteria [13,14,15,16,17,18,19,20]. Together they enrolled 151 patients with Parkinson’s disease, of whom 147 contributed analysable data, together with 36 healthy controls. Sample sizes ranged from 8 to 74 participants (median 16), reflecting the exploratory character of this literature. Study characteristics are summarized in Table 1.

The designs were heterogeneous. One study was a three-arm randomized controlled trial with computer-generated allocation concealed in sealed opaque envelopes [16]; one was a random-order crossover study comparing verum acupuncture with covert and overt placebo [15]; one was a non-randomized two-group study in which participants were allocated according to their own preference [18]; two were single-arm interventional studies, one longitudinal over eight weeks [17] and one within-participant against tactile stimulation [19]; and two were controlled cross-sectional comparisons between patients and healthy controls [13,14,20], one of which is described across two reports.

Three features were nonetheless common across the included studies. First, acupoint selection converged: GB34 (Yanglingquan) was used in five of the seven studies, alone or within a multi-point prescription, and was the only point shared by protocols developed independently in different countries. Scalp acupuncture over motor areas, DU20, GB20 and LR3 were the most frequent additional points. Second, image acquisition was homogeneous: all seven studies used 3-Tesla scanners with echo-planar BOLD sequences, most analyzing resting-state data through the fractional amplitude of low-frequency fluctuations, degree centrality or seed-based functional connectivity. Third, the regions reported as modulated overlapped substantially despite the differing protocols, centring on the precentral gyrus, prefrontal cortex, thalamus, cerebellum, insula, cingulate cortex and putamen.

The clinical outcome measures, by contrast, were heterogeneous, and the domain in which improvement was reported varied with the protocol: pain and its affective components in the study targeting pain [18]; gait parameters measured by inertial units in the scalp acupuncture study [19]; tremor and UPDRS-II and III scores in the randomized trial [16]; and motor performance of the affected hand in the crossover placebo study [15]. No two studies used the same combination of acupuncture protocol, assessment schedule and outcome instrument. This is the principal reason no quantitative synthesis was attempted.

### 3.3. Relatedness of the Included Studies

The included studies are not fully independent of one another, and the distinction between reports and studies matters here. Three of the eight included reports [13,14,17] originate from the same research group and are registered under a single trial registration (KCT0001122, “Development of Acupuncture Treatment for Patients with Parkinson’s disease”). Two of them [13,14] describe the same sample of 12 patients with Parkinson’s disease and 12 healthy controls undergoing acute GB34 stimulation, analyzed with different emphases—the earlier report examining global neural responses including the substantia nigra, caudate, thalamus and putamen, and the later one the between-group contrast in the prefrontal cortex and precentral gyrus. In accordance with PRISMA 2020 and the Cochrane Handbook, they are treated throughout as a single study reported twice, and data were extracted from both reports. The third [17] describes a separate eight-week longitudinal protocol in a smaller sample under the same registration; although its paradigm and observation period differ, participant overlap with the former cannot be excluded from the information published.

Two consequences follow. The seven included studies are reported in eight publications, and the effective number of independent investigations is therefore smaller than a count of reports would suggest. And any convergence of findings between the studies originating in this programme must be read as partly reflecting a shared protocol, sample frame and analytic tradition, rather than as independent replication. The synthesis below is reported accordingly.

### 3.4. Participants, Comparators and Allocation

Eligibility criteria were consistent across studies. All required a diagnosis of idiopathic Parkinson’s disease established by a neurologist, three applying the UK Parkinson’s Disease Society Brain Bank criteria explicitly, and all excluded atypical parkinsonism, other neurological disorders, and contraindications to magnetic resonance imaging. Additional criteria appeared in individual studies: right-handedness [16,19], mild-to-moderate disease severity defined by a Hoehn and Yahr stage below 4 [16,19], preserved cognition on the Mini-Mental State Examination [16,18], stable antiparkinsonian medication for at least two weeks [20], and pain of parkinsonian origin as an entry requirement in the study addressing pain [18].

Five comparator models were used: acupuncture against no treatment [18]; true against sham acupuncture and against a waiting list [16]; verum against covert and overt placebo [15]; acupuncture against tactile stimulation within the same participants [19]; and patients against healthy controls [13,14,20]. Only one study allocated participants by a randomized procedure with concealment [16]. In one, participants chose their own group [18]; in one, the order of conditions rather than the allocation of participants was randomized [15]; in two, there was no comparator group at all [17,19]; and in two, group membership was determined by disease status and was therefore not an allocation in any meaningful sense [13,14,20]. This distribution is the single most important constraint on what the present evidence can support, and it is returned to in Section 3.7 and Section 4.

### 3.5. Interventions and Acupoint Selection

GB34 was the most consistently used acupoint, appearing in five of the seven studies—alone [13,14,15,20], with LR3 [17], or within a wider prescription [18]. It was the only point common to protocols developed independently in Taiwan and South Korea. Needle gauge was similar throughout (0.25–0.30 mm diameter, 40 mm length), as was insertion depth at the point (approximately 1.0–1.5 cm), and where stimulation was specified, it was manual bidirectional rotation at 1 Hz to elicit the DeQi sensation.

Beyond GB34, the protocols diverged. Scalp acupuncture over the motor area, the chorea–tremor controlled area, and the balance area was used in one study [19] and the chorea–tremor controlled zone together with DU20 and GB20 in another [16], both with oblique subcutaneous insertion to 2.0–3.0 cm rather than perpendicular needling. One study delivered 120 Hz electroacupuncture followed by semi-individualized manual acupuncture selected according to each patient’s symptoms [17], so that the intervention was not uniform across participants within that study. Treatment intensity ranged from a single session lasting minutes [13,14,15,20] to 16 sessions over 10–14 weeks [18] and 24 sessions over 12 weeks [16]. This variation in point prescription, stimulation mode and cumulative dose means that no component of the intervention can be isolated as responsible for any observed effect.

### 3.6. Imaging Protocols, Assessment Schedules and Outcome Measures

Imaging was the most homogeneous element of this literature. All seven studies used 3-Tesla scanners with echo-planar BOLD acquisition. Analytical approaches were nonetheless varied: task-based or block-design contrasts in the acute studies [13,14,15], resting-state fractional amplitude of low-frequency fluctuations and degree centrality [19,20], regional homogeneity [17], and a combination of degree centrality, regional homogeneity and amplitude of low-frequency fluctuations [16]. Statistical thresholding differed materially and is relevant to the interpretation: two studies applied cluster-level correction [16,19], while the earliest study reported contrasts at an uncorrected threshold with a 10-voxel extent [15].

Assessment schedules ranged from a single acute session [13,14,15,20] to eight weeks with an eight-week follow-up [17] and to reassessment up to three months after the end of treatment [18]. Clinical instruments were correspondingly varied and included the UPDRS [16,17,18,20], the King’s Parkinson’s Disease Pain Scale and a visual analogue scale [18], the Beck Depression Inventory-II [17,18], the Parkinson’s Disease Sleep Scale-2 and the Parkinson’s Disease Questionnaire [18], the Hoehn and Yahr staging scale [16,20], instrumented gait analysis using inertial measurement units [19], and posturographic assessment [20].

Across studies, acupuncture was associated with modulation of a recurring set of regions: the precentral gyrus and primary motor cortex, prefrontal cortex, thalamus, cerebellum, insula, cingulate cortex and putamen. The clinical domain in which change was reported mapped onto the protocol rather than onto a single common effect: pain and its affective components engaged somatosensory cortex and insula [18]; tremor and motor scores engaged the cerebello-thalamo-cortical circuit [16]; gait engaged frontal and cerebellar regions indexed by the fractional amplitude of low-frequency fluctuations and degree centrality [19]; and cognitive and affective measures engaged default-mode and limbic regions [16,17].

### 3.7. Risk of Bias

Per-study, per-domain judgements are presented in Table 2a,b. No study was at low or moderate overall risk of bias.

Both studies with a randomized element were judged to be at high overall risk. In the three-arm randomized controlled trial [16] the randomization process was adequate—a computer-generated sequence with allocation concealed in sealed opaque envelopes—and outcome assessors and the imaging analyst were blinded throughout; the judgement was driven by missing outcome data, since 6 of 41 randomized participants (15%) were not analyzed, all of them from the sham and waiting-list arms and none from the verum arm, two of them withdrawing on the grounds of poor efficacy, with no intention-to-treat analysis reported. In the crossover study [15] the order of the three conditions was randomized and counterbalanced, and blinding was formally assessed using Bang’s index, which showed that nine of ten participants identified the verum condition as real; the judgement was driven by the absence of any washout period between three nine-minute conditions administered within a single session, and by BOLD contrasts thresholded without correction for multiple comparisons.

Among the five non-randomized studies, four were judged to be at serious and one at critical overall risk. The critical judgement [18] follows from the allocation procedure: participants who wished to receive acupuncture were assigned to the intervention group and those who declined became controls, so that expectancy, motivation and help-seeking behaviour are confounded with allocation and are prognostic for a self-reported pain outcome, with no adjustment reported and no blinded outcome assessment. The serious judgements arise from three recurring features: the absence of a concurrent control group in the single-arm designs [17,19]; the cross-sectional character of the patient-versus-control comparisons [13,14,20], which cannot support causal inference about a therapeutic effect; and, in one study [19], the exclusion of 4 of 12 enrolled participants after the intervention, two of them on the basis of a post-intervention variable, leaving an analyzed sample below the size the authors’ own power calculation had specified.

These judgements are carried into Section 4. The most confident inferences available from this evidence base rest on the single trial with concealed allocation and on the two studies employing a sham or placebo condition, and no included study is adequately powered to separate specific from non-specific effects.

**Table 2:** (**a**) Risk of bias in the two studies with a randomized element, assessed with RoB 2 [11]. (**b**) Risk of bias in the five non-randomized studies, assessed with ROBINS-I [12].

**(a)**									
**Study**	**D1**	**D1b**	**D2**	**D3**	**D4**	**D5**	**Overall**	**Principal Driver of the Judgement**	
Li et al., 2018 [16]	Low	n/a	Some concerns	High	Low	Some concerns	High	Six of 41 (15%) were not analyzed, all from the sham and waiting-list arms and none from the verum arm, two withdrawing due to poor efficacy; no intention-to-treat analysis	
Chae et al., 2009 [15]	Low	High	Some concerns	Low	Some concerns	Some concerns	High	Three conditions administered in one session with no washout; BOLD contrasts uncorrected for multiple comparisons; blinding not achieved for verum (Bang’s index 0.80)	
**(b)**									
**Study**	**C**	**S**	**CI**	**D**	**MD**	**MO**	**SR**	**Overall**	**Principal Driver of the Judgement**
Yu et al., 2019 [18]	Critical	Moderate	Low	Moderate	Moderate	Serious	Moderate	Critical	Groups formed by patient preference, so expectancy and help-seeking behaviour are confounded with allocation and prognostic for a self-reported pain outcome; no adjustment and no blinded assessment
Sun et al., 2022 [19]	Moderate	Serious	Low	Moderate	Serious	Low	Moderate	Serious	Four of 12 participants excluded after the intervention, two based on a post-intervention variable, leaving an analyzed sample below the authors’ own power requirement; condition order fixed
Yeo et al., 2012/2014 [13,14]	Serious	Moderate	Low	Moderate	Low	Moderate	Moderate	Serious	Cross-sectional comparison between patients and healthy controls; the design cannot support causal inference about a therapeutic effect
Yeo et al., 2018 [17]	Serious	Moderate	Low	Serious	Moderate	Serious	Moderate	Serious	Single-arm design with no control condition and no blinding; subjective primary outcomes; semi-individualized intervention not uniform across participants
Lee et al., 2024 [20]	Serious	Moderate	Low	Moderate	Low	Moderate	Moderate	Serious	Cross-sectional design with an acute stimulation challenge, no sham condition and no therapeutic follow-up

D1, randomization process; D1b, carry-over and period effects (crossover extension); D2, deviations from intended interventions; D3, missing outcome data; D4, measurement of the outcome; D5, selection of the reported result. C, confounding; S, selection of participants; CI, classification of interventions; D, deviations from intended interventions; MD, missing data; MO, measurement of outcomes; SR, selection of the reported result.

## 4. Discussion

### 4.1. Principal Findings

Across the seven included studies, acupuncture was associated with modulation of activity in motor, limbic and cerebello-thalamo-cortical circuits in patients with Parkinson’s disease, and in several studies, this modulation occurred alongside improvement in pain, gait or motor performance. The convergence is notable because it emerged from protocols developed independently in Taiwan, China, and South Korea, using different analytical pipelines and different clinical instruments: the precentral gyrus and primary motor cortex, prefrontal cortex, thalamus, cerebellum, insula, cingulate cortex, and putamen recur across studies that share almost nothing else.

GB34 is the most consistent element of that convergence. It was used in five of the seven studies and was repeatedly associated with the engagement of motor cortical and subcortical regions, including the putamen and primary motor cortex in the crossover placebo study [15] and the precentral gyrus in the cross-sectional comparison [14]. Multi-point protocols incorporating DU20, GB20, LR3 and scalp motor areas were associated with broader modulation extending to cognitive and cerebello-thalamo-cortical networks [16,17,19], which suggests that point selection may influence the distribution of neural effects. That suggestion is a hypothesis generated by comparison across studies, not a finding within any of them, since no included study compared point prescriptions directly.

Two studies are informative about specificity, and both qualify the picture. The crossover study contrasted verum acupuncture with a covert placebo and found activation of the primary motor cortex and putamen attributable to needling rather than to expectancy [15]; but its three conditions were administered in a single session without a washout, and its contrasts were not corrected for multiple comparisons. The randomized trial contrasted true with sham acupuncture and with a waiting list, and reported a tremor-specific effect [16]; nevertheless, blinding success was never evaluated, and all six unanalysed participants came from the two control arms. The evidence is therefore compatible with a specific neuromodulatory effect, although only two of the seven studies included a sham or placebo condition capable of testing this, and neither was adequately powered to do so.

What the evidence does not establish is whether the modulation is specific to needling, whether it is durable, and whether it translates into clinically meaningful benefit. Answering those three questions requires trials designed for the purpose, a point taken up in Section 4.4.

### 4.2. Clinical Implications

Parkinson’s disease is characterized not only by motor dysfunction but also by pain, gait impairment, cognitive and affective symptoms, and reduced quality of life, and the non-motor burden is frequently the greater one. The studies reviewed here address several of these domains, and the alignment they show between neurofunctional change and clinical change is what makes them interesting: pain relief accompanied by the modulation of the somatosensory cortex and insula [18], gait improvement accompanied by frontal and cerebellar modulation [19], and tremor reduction accompanied by the engagement of the cerebello-thalamo-cortical circuit [16].

That alignment is consistent with, but not sufficient to establish, a specific neurobiological mechanism; the available designs cannot separate specific from non-specific effects. Nor are these results sufficient to support a change in clinical practice. Seven small studies, one of which used concealed randomized allocation and none of which was at low risk of bias, do not constitute a basis on which to recommend acupuncture as part of the routine multidisciplinary management of Parkinson’s disease, however plausible the mechanism and however favourable the safety profile reported.

What these results do provide is a specification for the trial that would settle the question: the outcome measures that track neurofunctional change, the control conditions capable of isolating a specific effect, and the sample sizes such a trial would require. That is a more useful contribution than a premature recommendation, and it is the contribution we claim.

### 4.3. Limitations

The principal limitation of this review is the heterogeneity of the evidence it synthesizes. The included studies differ in design—one randomized controlled trial, one randomized-order crossover study, one non-randomized two-group study, two single-arm interventional studies and two controlled cross-sectional comparisons—and also in allocation procedure, acupoint prescription, stimulation parameters, treatment duration, assessment schedule and imaging analysis pipeline. No two studies share the same combination of protocol and outcome instrument. Quantitative synthesis was therefore not possible, effect magnitudes cannot be compared directly between studies, and the convergence reported in Section 3 should be read as qualitative agreement about which circuits are engaged rather than as agreement about the size of any effect.

The evidence base is also small and of low methodological quality. Sample sizes ranged from 8 to 74 participants, with a median of 16; no study was at low or moderate risk of bias; only one employed concealed randomized allocation; and only two included a sham or placebo condition. As set out in Section 3.3, three of the eight included reports originate from a single research programme under a shared trial registration, so the apparent independence of the evidence is smaller than the number of studies suggests.

Three further limitations concern the review process itself. This review was not prospectively registered, which limits the assurance that can be given against selective reporting of its own methods and outcomes. EMBASE could not be searched directly, as no institutional access was available, and coverage of EMBASE-indexed records was therefore partial, through CENTRAL. Eligibility was restricted to reports published in English or Portuguese; given the volume of acupuncture research published in Chinese, this restriction may have excluded relevant studies and is a material limitation in this field rather than a formality.

Finally, this review was restricted to peer-reviewed literature reporting results, and study protocols and conference abstracts were excluded. It may therefore be subject to publication bias, since unpublished studies with null findings would not have been identified. This risk was mitigated, but not removed, by searching trial registers alongside bibliographic databases; the registers returned twenty ongoing or completed trials for which no results have yet been published, which is itself an indication of how much of this literature remains unreported.

### 4.4. Requirements for Future Research

The design of a confirmatory trial follows directly from the weaknesses documented in Table 2a,b. Allocation should be randomized with concealment, and blinding success should be measured rather than assumed—the one included study that measured it found that nine of ten participants correctly identified verum acupuncture [15], which suggests that credible sham control in this field is harder than it is usually reported to be. Outcome assessment should be blinded, and the primary clinical outcome should be pre-specified rather than selected from a panel of instruments after the fact. Attrition should be reported by arm and analyzed by intention to treat; in the one randomized trial available, all attrition fell in the control arms, and two participants withdrew citing poor efficacy, a pattern that biases the comparison in a direction favourable to the intervention.

On the imaging side, acquisition and analysis pipelines should be harmonized and pre-specified, statistical thresholds should be corrected for multiple comparisons, and sample size should be calculated for the neuroimaging outcome rather than for the clinical one, since the former is typically the more demanding. Follow-up should be long enough to distinguish an acute response from a cumulative neuroplastic effect; among the included studies the observation period ranged from a single session to four months, and the two designs answer different questions. Finally, prospective registration with a pre-specified analysis plan would address the single limitation that recurs in every study reviewed here.

## 5. Conclusions

This review synthesized the available functional MRI evidence on the neural effects of acupuncture in Parkinson’s disease. Across seven studies published between 2009 and 2024, acupuncture was consistently associated with the modulation of motor, sensory, cognitive and affective brain networks, including the precentral and prefrontal cortices, basal ganglia, thalamus, cerebellum, insula and components of the default-mode network. In several studies these neurofunctional changes were accompanied by improvement in motor performance, gait parameters or pain.

The consistency of the regions implicated, across protocols developed independently in different countries, is the most notable finding of this synthesis. GB34 in particular was used in five of the seven studies and was repeatedly associated with the engagement of motor cortical and subcortical circuits. That consistency is a reason to pursue the question further; it is not, in itself, evidence of clinical efficacy.

The limits of this evidence are as important as its content. The included studies are small, methodologically heterogeneous, and uniformly at high, serious or critical risk of bias; only one used concealed randomized allocation, only two included a sham or placebo condition, and three of the eight reports originate from a single research programme. These findings should therefore be regarded as hypothesis-generating. They are not sufficient to establish clinical efficacy, to distinguish specific neuromodulatory effects from expectancy or other non-specific effects, or to support a change in current clinical practice.

What this synthesis does provide is a specification for the research that would resolve the question. A confirmatory trial would require concealed randomized allocation, a credible sham condition with formal assessment of blinding success, a sample adequately powered for the neuroimaging outcome, a standardized acupuncture protocol, blinded clinical outcome assessment, imaging acquisition and analysis pipelines harmonized across sites, correction for multiple comparisons, follow-up long enough to establish durability, and prospective registration with a pre-specified analysis plan. Until trials of that kind are conducted, functional MRI will continue to describe what acupuncture does to the brain in Parkinson’s disease without establishing what it does for the patient.

## Figures and Tables

**Table 1:** Characteristics of the seven included studies.

Study, Country, Design	Participants	Intervention and Comparator	Assessment and Imaging	Principal Findings
Chae et al., 2009 [15] South Korea Random-order crossover, single session	10 patients with idiopathic PD Other neurological diseases excluded No losses	Left GB34; 0.25 × 40 mm needle to 10 mm, manual rotation at 1 Hz Three 9 min conditions in randomized, counterbalanced order: verum, covert placebo (non-penetrating sham needle), and overt placebo (identical sham, declared inert) No washout between conditions	Acute effect within one session Motor function of the affected hand Blinding quantified with Bang’s index 3 T, gradient-echo EPI T2*-weighted BOLD (TR 3 s, TE 25 ms); threshold Z > 2.81, *p* < 0.005 uncorrected, 10-voxel extent	Verum versus covert placebo engaged right primary motor cortex, left middle frontal gyrus, putamen and precuneus; the placebo contrast engaged anterior cingulate, superior frontal and superior temporal gyri Motor function of the affected hand improved after verum Nine of ten participants identified verum as real
Yeo et al., 2012 [13] and 2014 [14] South Korea Controlled cross-sectional, block design with sham One study reported twice	12 patients with idiopathic PD and 12 healthy participants Atypical parkinsonism, other neurological, medical and psychiatric conditions excluded	Right GB34; 0.25 × 40 mm needle to 1 cm, bidirectional rotation at 1 Hz Alternating stimulated and non-stimulated blocks; blunt non-penetrating sham needle as control	Single session, acute effect only No longitudinal clinical outcome 3 T Philips, EPI Registered KCT0001122 (30 May 2014)	2012 report: GB34 stimulation engaged substantia nigra, caudate, thalamus and putamen 2014 report: patients showed greater activation than healthy participants in prefrontal cortex and precentral gyrus, predominantly in the left hemisphere Design cannot attribute group differences to the intervention
Li et al., 2018 [16] China Three-arm randomized controlled trial, 12 weeks	41 patients with PD with tremor (UK Brain Bank criteria), H&Y ≤ 4, aged 45–80, MMSE ≥ 24, BDI ≤ 4 True acupuncture n = 14, sham n = 14, waiting list n = 13 6 of 41 (15%) not analyzed, all from the sham and waiting-list arms	DU20, GB20 and the chorea–tremor controlled zone; stainless steel needles to 2.0–3.0 cm, twirling every 10 min, 30 min per session, twice weekly for 12 weeks Sham: 0.2 cm depth, 0.5 cun from the true point, no manipulation Waiting list untreated All arms continued levodopa	Before and after the 12-week course UPDRS II and III with blinded assessors; imaging analyst blinded to allocation 3 T with 8-channel head coil, T2*-weighted gradient-recalled EPI; DC, ReHo and ALFF Allocation by computer-generated sequence in sealed opaque envelopes	UPDRS II and III improved in the true-acupuncture arm, with a specific effect on the tremor sub-score Modulation of the cerebello-thalamo-cortical circuit (cerebellum, thalamus, motor cortex) and of cognitive regions (default-mode network, visual areas, insula, prefrontal cortex) Blinding success was not evaluated
Yeo et al., 2018 [17] South Korea Single-arm pre–post, 8 weeks	10 outpatients with PD from a single hospital No control group	120 Hz electroacupuncture at right GB34 and LR3 for 15 min, followed by semi-individualized manual acupuncture selected according to each patient’s symptoms; twice weekly for 8 weeks Intervention therefore not uniform across participants	Baseline, immediately after 8 weeks, and 8 weeks later UPDRS sub-scores and depression scores, unblinded 3 T fMRI; ReHo Registered KCT0001122, shared with [13,14]	UPDRS sub-scores and depression scores fell after treatment and remained stable at follow-up Increased neural response in thalamus, cingulate and anterior cingulate, lingual and parahippocampal gyri, lateral globus pallidus, mammillary body, middle temporal gyrus, cuneus and fusiform gyrus Change in thalamic signal correlated with change in UPDRS-III
Yu et al., 2019 [18] Taiwan Non-randomized two-group study; allocation by patient preference	16 patients with idiopathic PD and PD-related pain (KPPS > 0) Acupuncture n = 9, control n = 7 Groups formed according to each patient’s own wish; previous acupuncture within 3 months excluded	GV20, GB34 and extra point 77.18; 0.30 × 40 mm needles to 5–10 mm, DeQi elicited for 20 s, 30 min per session, one to three sessions weekly, 16 sessions in total Controls continued analgesic therapy alone, with no sham	Baseline, after 16 sessions (controls reassessed at 10–14 weeks), and up to 3 months after treatment KPPS, UPDRS, BDI-II, PDSS-2, PDQ, MMSE, VAS; no blinded assessment 3 T, T1-weighted MPRAGE and rs-fMRI; seed-based functional connectivity	KPPS and UPDRS total scores fell by 46.2% and 21.6% respectively Increased connectivity in four pairs involving middle temporal gyrus, precentral and postcentral gyri, supramarginal gyrus and insular cortex Change in left middle frontal–right precentral connectivity correlated with change in KPPS (R = −0.698, *p* = 0.037) Pain relief persisted up to 3 months
Sun et al., 2022 [19] China Single-arm within-participant pilot; scalp acupuncture versus tactile control	12 right-handed patients with mild-to-moderate PD (H&Y < 4) enrolled; 8 analysed Four excluded after the intervention: two for differing needle sensation between conditions, two for head motion exceeding 2 mm or 2° An a priori power calculation had specified 12	Scalp acupuncture over the upper fifth of the motor area, the chorea–tremor controlled area and the balance area; 0.25 × 40 mm needles inserted subcutaneously at 15° to 2.0–3.0 cm, 20 min Tactile stimulation as the control condition, always administered first	Two resting-state scans: after tactile stimulation and after needle removal Gait recorded by IMU at 100 Hz; needle sensation rated 0–3 3 T Siemens Magnetom Skyra; fALFF and DC, cluster-corrected Single-blind	Stride length, maximum ankle height, maximum ankle horizontal displacement, gait speed and shank range of motion all increased (*p* < 0.05) fALFF increased in left middle frontal gyrus and DC in left cerebellum; fALFF decreased in left inferior parietal lobule Right limb swing correlated positively with both fALFF clusters Condition order fixed, not randomized
Lee et al., 2024 [20] South Korea Controlled cross-sectional study with acute stimulation	74 participants: 50 patients with idiopathic PD (UK Brain Bank criteria, stable medication for ≥2 weeks) and 24 age- and sex-matched healthy controls Secondary parkinsonism, Parkinson-plus syndromes, major medical and psychiatric illness excluded No losses	Bilateral GB34; non-magnetic 0.25 × 40 mm needles to 1.0 cm, DeQi by bidirectional rotation at 1 Hz, needles retained during the second acquisition Comparator: the pre-stimulation scan and the healthy control group; no sham	Two resting-state acquisitions, at rest and with needles retained Cross-sectional UPDRS, H&Y, disease duration and Balance Master posturography 3 T; zfALFF, seed-based functional connectivity, voxel-based morphometry, mixed-effects models with group × intervention interaction	Reduced spontaneous activity in right cerebellar Crus II in patients, with altered connectivity to parahippocampal, supramarginal and superior frontal regions GB34 stimulation reduced zfALFF in right inferior occipital gyrus in patients and in bilateral lingual gyri in controls, with a significant group × intervention interaction in left calcarine cortex No therapeutic follow-up

Abbreviations: ALFF, amplitude of low-frequency fluctuations; BDI-II, Beck Depression Inventory-II; DC, degree centrality; EPI, echo-planar imaging; fALFF, fractional ALFF; HC, healthy controls; H&Y, Hoehn and Yahr stage; IMU, inertial measurement unit; KPPS, King’s Parkinson’s Disease Pain Scale; MMSE, Mini-Mental State Examination; PD, Parkinson’s disease; PDQ, Parkinson’s Disease Questionnaire; PDSS-2, Parkinson’s Disease Sleep Scale-2; ReHo, regional homogeneity; rs-fMRI, resting-state functional magnetic resonance imaging; UPDRS, Unified Parkinson’s Disease Rating Scale; VAS, visual analogue scale.

## Data Availability

No new data were created in this study. All data analyzed are contained within the publications cited in the reference list, which are publicly available. The data extraction forms, the screening log, and the completed risk-of-bias assessments are available from the corresponding author upon reasonable request.

## Supplementary Materials

The following supporting information can be downloaded at: https://www.mdpi.com/article/10.3390/healthcare14203457/s1, Table S1: Database-specific search strategies, filters, dates of execution and number of records retrieved.
