# Supplementary material for: Neuroimaging Evidence of Acupuncture Effects in Parkinson’s Disease: A Systematic Review of fMRI-Based Clinical Studies
Source: Healthcare (Basel). 2026 Oct 15;14(20):3457. doi: 10.3390/healthcare14203457 (PMC13704738; doi:10.3390/healthcare14203457)
Supplement: Supplementary file 1 [file healthcare-14-03457-s001.zip › healthcare-4485772-supplementary.pdf]

Supplementary Table S1

Database-specific search strategies, filters, dates of execution and number of records retrieved

Manuscript: Neuroimaging Evidence of Acupuncture Effects in Parkinson's Disease: A Systematic Review of fMRI-Based Clinical Studies.

Pereira CR, Greten HJ, Santos R, Reis AM, Ramos B, Santos MJ, Machado J, Pereira F, de Oliveira NM, Criado MB.

1. Initial search (as executed, 1 March – 30 April 2025)

Search string: (Parkinsonian disorders OR Parkinson OR Parkinson Disease OR Parkinson's Disease OR Parkinsonism) AND (Functional Magnetic Resonance OR fMRI) AND (Acupuncture)<br>Filters applied: publication date 2013–2023; language English or Portuguese; species human; article type randomized controlled trial.

Sources and records retrieved:

Source	Records	Outcome
PubMed (including MEDLINE)	0	No records returned with the filters applied
ScienceDirect	1	Review article (doi:10.1016/S0254-6272(15)30164-3); excluded
The Cochrane Library	8	4 duplicates of PubMed-indexed records retrieved within the Cochrane Library; 1 study protocol (doi:10.3390/ijerph20031776); 1 out of scope (doi:10.1002/mdc3.12744); 2 retained
Cochrane Central Register of Controlled Trials (CENTRAL)	7	Trial-register entries only, with no associated publications
SciELO	0	—
EMBASE	—	Not searched directly (no institutional access); EMBASE-indexed records are covered by the Cochrane Library

Totals: 9 records from bibliographic databases (ScienceDirect 1, Cochrane Library 8) and 7 from trial registers (CENTRAL). After removal of 4 duplicates and exclusion of 3 records (1 review, 1 study protocol, 1 out of scope), two reports met the criteria applied at that stage: Yeo et al., 2014 (doi:10.1186/1472-6882-14-336) and Li et al., 2018 (doi:10.3389/fnagi.2018.00206).

Note. Duplicates refer to PubMed-indexed records that appeared within the Cochrane Library results. PubMed itself returned no records with the filters applied. Run without the randomized-controlled-trial filter, PubMed returned 23 records, of which 6 appeared potentially eligible on abstract screening (patients with Parkinson's disease, acupuncture, fMRI outcome). The Cochrane Library contributed one further potentially eligible record not indexed in PubMed. With the RCT filter alone, PubMed returned 1 record.

2. Updated search (15 September 2026, after the eligibility criteria were broadened)

Search string (PubMed syntax; adapted to the controlled vocabulary and field tags of each database):<br>("Parkinson Disease"[Mesh] OR Parkinson*[tiab] OR parkinsonism[tiab])<br>AND ("Magnetic Resonance Imaging"[Mesh] OR fMRI[tiab] OR "functional magnetic resonance"[tiab] OR BOLD[tiab] OR "resting state"[tiab])<br>AND ("Acupuncture Therapy"[Mesh] OR acupuncture[tiab] OR electroacupuncture[tiab] OR acupoint*[tiab] OR needling[tiab])<br>Filters applied: no date limit; no publication-type limit; humans; English and Portuguese.

3. Records retrieved per source (updated search)

Database / source	Records retrieved	Date of execution	Notes

PubMed / MEDLINE	10	15 September 2026	Searched without filters; MEDLINE fully covered
Cochrane Library (including CENTRAL)	32	15 September 2026	12 journal records and 20 trial-register entries (ICTRP, ClinicalTrials.gov, ChiCTR, CRIS/KCT, ITMCTR)
ScienceDirect	1	15 September 2026	Re-run without filters; no new record. The single record is a review and was excluded
SciELO	0	15 September 2026	Re-run without filters; no records
EMBASE	-		Searched with the same strategy and without filters
Citation searching (reference lists of included reports and of relevant reviews)	1 report	1 March – 30 April 2025	One report identified as potentially eligible and included (Sun et al., 2022), retrieved by neither database. The number of individual citations screened by hand was not recorded prospectively; this is acknowledged in Section 4.3
**Total identified (databases and registers)**	**43**	—	23 from bibliographic databases and 20 from trial registers; 4 duplicates removed before screening

All databases were searched with the same strategy and without filters. The reference lists of all included reports and of relevant reviews were additionally searched by hand; studies identified in this way are reported in the second arm of the PRISMA flow diagram (Figure 1). This review was not prospectively registered; the protocol was defined a priori by the review team and is available from the corresponding author on request.
